# Adapting Nursing Care During the COVID-19 Pandemic: Staff Nurses' Experiences, Lessons Learned, and Implications for Nursing Management

**DOI:** 10.1155/jonm/6178630

**Published:** 2025-05-29

**Authors:** Nahed Alquwez

**Affiliations:** Department of Nursing Administration and Education, College of Nursing, Shaqra University, Al Dawadmi, Saudi Arabia

**Keywords:** COVID-19, nursing care, nursing practice, qualitative study, Saudi Arabia

## Abstract

The COVID-19 pandemic experiences of nurses presented many challenges to all aspects of society, including healthcare. Nursing care must adapt to these changes amidst this pandemic to ensure quality nursing care. Thus, exploring the changes in nursing care for COVID-19 patients is essential in understanding how nurses adapt and adjust during a pandemic. This study explored the experiences of staff nurses in providing nursing care during COVID-19 and how they adapted their nursing care to the situation during the pandemic. Using the qualitative phenomenological design, utilizing a semantic thematic approach, 15 nurses in COVID-19 units in government hospitals in Saudi Arabia participated in this study. The study conducted in-depth face-to-face and Zoom interviews from September to December 2021. The analysis followed the steps of the semantic thematic approach. This study showed the evolving nursing care for COVID-19 patients in six major themes: emotional instability, learning, re-learning, and unlearning nursing care, resourcefulness in nursing practice, accepting and adjusting nursing roles, living with COVID-19, and spiritual belief and care. It was uncovered that nurses experienced emotional instability during the pandemic. The pandemic also provided opportunities for nurses to learn new knowledge and skills on COVID-19 prevention, protection, and intervention. They further re-learned basic knowledge on infection control and unlearned nursing practices that were not correctly performed before the pandemic.

## 1. Introduction

Nurses played a frontline role in mitigating the spread of COVID-19 by implementing infection control protocols, conducting triage assessments, administering vaccinations, and educating patients on hygiene and preventive behaviors [1]. Their responsibilities expanded significantly during the pandemic, requiring rapid adaptation and resilience in highly dynamic healthcare settings. Nurses play a vital part in preventing and mitigating the spread of COVID-19 through activities such as infection control, vaccination administration, patient education, and care coordination [2–4]. These roles became crucial in the evolving healthcare landscape during and after the pandemic, underlining the need to study their lived experiences in such contexts. Nurses live with the idea of being exposed to an unprecedented health hazard [1]. The pandemic demanded a high level of knowledge and skills among nurses and more training to cope with the demand for care required for patients with COVID-19 [2]. However, studies mentioned that nurses provide care to patients with COVID-19 without adequate knowledge.

Also, most available data show that nurses encountered physical and emotional problems while caring for COVID-19 patients [3–5]. Balay-Odao et al. reported that nurses experience psychological problems during COVID-19 [6]. Another study has shown that nurses experience stress, fatigue, dilemma, and issues of donning and doffing personal protective equipment (PPE) in the provision of nursing care during this pandemic [7]. During a crisis, establishing well-planned nursing care is not easy. Activities that enhance the training and skills of nurses are essential to align competencies with patient acuity levels, particularly as many nurses were deployed to high-dependency or critical care settings during the COVID-19 pandemic, necessitating rapid upskilling.

Previous studies have reported the lived experiences of nurses in Arab countries during the COVID-19 pandemic. For example, a multicountry qualitative study involving nurses from Bahrain, Kuwait, Oman, Saudi Arabia, and the United Arab Emirates (UAE) described their experiences caring for COVID-19 patients as filled with doubts and chaotic challenges. Despite these difficulties, the nurses progressed toward professional resilience and managed to achieve an optimum level of nursing care [8]. Another study focused on the experiences of frontline nurses in Qatar during the pandemic. It highlighted various challenges that affected their physical, emotional, and psychological well-being, including the stress of adapting to a new environment, heavy workloads, the necessity of using PPE, fear, and witnessing patient suffering [9]. Additionally, these nurses reported that they survived the pandemic by adhering to extra safety measures, fostering teamwork and camaraderie, receiving strong social support, and adapting their eating habits. The resilience of these nurses was a significant focus of the study [9]. Furthermore, a qualitative study of nurses in Jordan during the COVID-19 pandemic described the various burdens they faced, including professional relationship stresses, personal challenges, environmental factors, physical symptoms, and emotional strains. These burdens significantly influenced the nurses' thoughts, feelings, and behaviors during the crisis [10]. Expatriate nurses working in the UAE during the pandemic, particularly those from the Philippines, faced several additional challenges. These included feelings of homesickness, the difficulty of being away from their families, cultural challenges, and the cancellation of their plans due to the pandemic [11].

However, despite the wealth of studies exploring the lived-experiences of nurses from Arab countries during the COVID-19 pandemic, research on the changes in nursing care amidst this pandemic is limited. Therefore, the evaluation of these changes is relevant and practical. Given the unprecedented challenges faced during the COVID-19 pandemic, this study offers novel insights into how nurses adapted their professional roles, coped with systemic limitations, and managed personal emotional burdens, which are areas that remain underexplored, particularly in the context of nurses working in Saudi Arabia.

Nurses must adapt their roles during this pandemic to protect themselves, their families, patients, and colleagues [12]. However, earlier studies have shown that hospital management lacks established protocols and guidelines [13]. Notably, the pandemic protocols and guidelines are also constantly changing and are thus confusing [4]. Siegfried et al. posited that access to information is essential during a pandemic [14]. Accurate information helps health nurses provide effective and quality patient care [15]. Moreover, if a person is aware of unreliable information sources, the quality of nursing care is not compromised [16]. Amidst this COVID-19 pandemic, nurses utilize the frequently changing guidelines and protocols to keep themselves well-versed in updated information [6]. During the pandemic's early period, nurses and other healthcare providers also lacked information on the pathophysiology, management, and intervention of COVID-19, thereby increasing psychological problems and insecurity [7]. Thus, acquiring evidence-based nursing practices is essential in promoting quality nursing care [17, 18].

Few evidence-based practices have been identified during the COVID-19 outbreak, and nurses must gain more information to provide quality nursing care. Globally, COVID-19 information floods various social media platforms [19], and nurses often use this information to increase their knowledge of this disease [15]. This information may have helped nurses in changing their care for COVID-19 patients. Changes in nursing care amidst this pandemic may occur to adapt to the volatile situations in the clinical settings and the rapidly evolving protocols to combat the pandemic. Nurses continuously discover and uncover information needed to adjust and adapt their clinical interventions as they provide care to COVID-19 patients. The COVID-19 pandemic led to significant shifts in nursing practices, including increased reliance on PPE, adoption of telehealth for noncritical consultations, stricter infection control protocols, and reassignments from general wards to critical care units. These changes altered nurses' daily routines, communication methods, and patient interaction styles.

Thus, exploring the changes in nursing care for nurses to COVID-19 patients is essential in understanding how nurses adapt and adjust during a pandemic. Nurses' preparedness during the COVID-19 pandemic is essential to understand, particularly in light of their critical role in frontline response efforts [6], mainly because COVID-19 is very contagious compared with other respiratory infection outbreaks (i.e., severe acute respiratory syndrome and MERS-CoV). However, there is limited information on the changes of care of nurses when providing nursing care to COVID-19 patients. Therefore, exploring how nursing care for COVID-19 has changed is relevant and essential. The study provides lessons from the nursing experiences during the pandemic and identifies practical implications for nursing management.

### 1.1. Aim

This study explored the experiences of staff nurses in providing nursing care during COVID-19 and how it evolved to adapt to the situation during the pandemic.

## 2. Materials and Methods

This study utilized the exploratory qualitative design to explore the changes in the nursing care of COVID-19 patients using reflective thematic analysis. The “Consolidated criteria for reporting qualitative studies” (COREQ) guided the reporting for this study [20]. Fifteen nurses participated in this study, including one Indian, eleven Filipinos, and three Saudis. When the study was conducted, the participants were employed in COVID-19 units of government hospitals in Riyadh, Al Kharaj, Al Dawadmi, Shaqra, and Quiyiyah. These nurses were between 27 and 40 years old and had 2–20 years of experience. They were recruited using a purposive sampling technique. The recruitment criteria included nurses who provided care for COVID-19 patients. Nurses unwilling to participate and did not want to share their experiences were excluded. Data saturation was achieved in the 14th participant, but a 15th participant was included to ensure no new codes would emerge. Braun and Clarke stated that data saturation is achieved when no new codes are identified during data collection and analysis [21]. The participants' characteristics are listed in Table 1.

### 2.1. Ethical Considerations

This study is part of a protocol approved by the “Research Ethics Standing Committee” of Shaqra University. In signing the consent, the participant was given full disclosure and information about their rights as study participants. To protect the identity of the respondents, the researcher used pseudonyms in the transcript file and data reporting. The researcher ensured that no participant identifiers were included in the transcript file. Names and places mentioned by the participants were replaced with the name X or the place X. The respondents were asked if they had concerns about the interview being recorded. The files and recordings were kept on the researcher's password-protected laptop and deleted once the study was concluded. In compliance with ethical research standards, the data will be retained for a minimum of 5 years.

### 2.2. Data Collection

The researcher conducted in-depth face-to-face and Zoom interviews from September to December 2021. The Zoom interviews were conducted for nurses who were unavailable for face-to-face interviews and for female participants who could not travel. The participants were identified from the endorsement of the researcher's friends. However, the researcher ensured that those potential participants met the inclusion criteria to be illegible for the study, thus ensuring they could contribute rich data about the nurses' experiences of caring for COVID-19 patients from the start of the pandemic. The researcher then approached the identified participants and explained the study's purpose and their participation rights before asking them to sign the written informed consent. The researcher and participant set the possible time based on the participant's schedule. The four Zoom interviews were digitally recorded, and the eleven face-to-face interviews were recorded using a recording device with the participants' permission. The primary question was, “Can you share your experience caring for a COVID-19 patient from the start of the pandemic?” Follow-up questions were based on the responses of the participants. The participants' answers were further explored to ensure the rigor of the data. The interview took about 40–70 min. After gathering data from in-depth face-to-face and Zoom interviews, the researcher compared the data quality, including the responses' depth, richness, and consistency. No significant differences in consistency were observed. After each interview, the researcher transcribed the data and generated initial codes, then returned the data to the participant for comments and corrections on the codes. The researcher continued to iterate this process until no new codes were developed.

### 2.3. Data Analyses

The data were transcribed into a Word file using five columns: the first column contained the participants' verbatim responses, the second column contained the extracted verbatim, the third column included the codes, the fourth column listed the subthemes, and the fifth column contained the themes. Data extraction, coding, and theme development were performed manually.

The researcher used the thematic approach in the data analysis. The first step is familiarization with the data. The researcher immersed himself in the data by reading it multiple times to understand the content. The researcher notes initial thoughts, patterns, and ideas that emerge. Then, the researcher revisits the data multiple times to deepen their understanding and ensure no important details are overlooked.

The second stage is the generation of initial codes. After familiarizing himself with the data, the researcher began generating initial codes. These short labels or phrases capture key data relevant to the research questions. Coding is an ongoing process, with researcher refining and adjusting codes as they explore the data, sometimes revisiting earlier segments to modify or refine their initial codes.

The third stage is searching for themes. After initial coding, the researcher organizes related codes into broader themes. Further, the researcher continuously moves between the data and the codes to refine and validate the themes, ensuring they reflect the data. After initial coding, the researcher revisits the data to ensure the themes are accurate and meaningful. If a theme is too broad or lacks specificity, it is adjusted. Themes are compared for overlap; if they are similar, they are merged. If a theme covers multiple ideas, it is split into more focused subthemes to provide a nuanced understanding of the data.

The fourth stage is reviewing themes. The researcher continually reviewed the themes in an iterative process. The researcher revisits the data and the initial codes to ensure the themes remain accurate, coherent, and consistent. This process ensures that the themes are comprehensive and nuanced. Themes may be revised, redefined, or discarded if the data do not adequately support them.

The fifth stage is defining and naming themes. After the researcher reviewed the themes, the researcher defined and named them. The researcher refined the themes of the entire dataset, ensuring that the themes represented the most significant patterns while accurately capturing the participants' perspectives. The last stage is writing the analysis. Writing the final analysis, the researcher continues to iterate on the themes and the data to maintain coherence. The researcher refines how the themes are presented, adds additional interpretations, or reconsiders the best way to communicate the complexity of the findings [22].

### 2.4. Rigor/Trustworthiness

Rigor or trustworthiness was ensured by observing the credibility, dependability, confirmability, transferability, and reflexivity criteria [23, 24]. The researcher ensured data credibility by synthesizing the interpretations and producing a final set of findings; the researcher asked two external qualitative researchers who are healthcare professionals to help ensure that the researcher's biases or perspective did not influence the data extracted, coded, and themes developed. The two external qualitative researchers separately evaluate the codebooks and compare their evaluations afterward. Any discrepancies were discussed with the researcher, and the appropriate codes were agreed upon. Also, member checking was performed to ensure that the themes developed were based on the participants' perspectives. Dependability was assured by reporting adequate and appropriate information about the participants in the study, offering context for the study's findings. Detailed participant information (like demographics, professional background, or experiences) allows readers to understand the perspectives represented and assess how consistently data collection methods were applied across participants. Also, dependability in this study was ensured through a transparent and well-documented research design, including detailed descriptions of the study's purpose, methods, and data collection procedures.

Additionally, rigorous data collection and analysis techniques were applied consistently across participants and settings, ensuring reliability and consistency throughout the research process. To ensure confirmability, the researcher conducted data extraction, coding, and theme identification in collaboration with two external qualitative researchers. Both external researchers independently coded and categorized the data, and any differences were discussed to reach a consensus. The researcher and the two external evaluators collaboratively developed the final themes, ensuring that the findings were grounded in the data and not influenced by the researcher's biases or assumptions. Triangulation was also used to check the validity of the findings. The researcher had a dialogue with the two external qualitative researchers to identify his assumptions and biases. Also, the researcher was mindful of his position, theoretical assumptions, and bias throughout the research process by keeping a research journal. To ensure transferability, the study provided rich, detailed descriptions of the research context, participants, and data collection process. This enables readers to assess the applicability of the findings to other settings or populations.

## 3. Results

**Figure 1:**
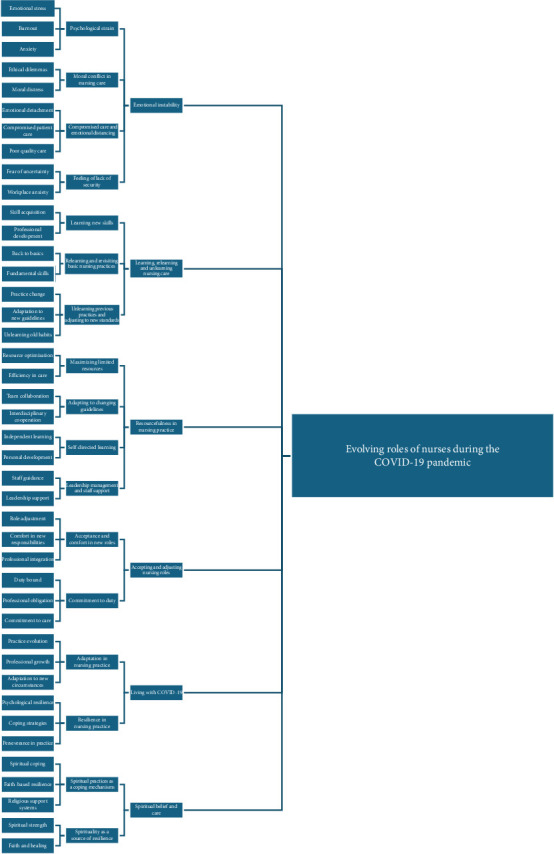
Themes and subthemes derived from the thematic analysis.

This study explored the evolving nursing care for COVID-19 patients presented by six themes: emotional instability, learning, relearning, and unlearning nursing care, resourcefulness in nursing practice, accepting and adjusting nursing roles, living with COVID-19, and spiritual belief and care (Figure 1).

This study explored the changes in nursing care for COVID-19 patients, which were presented by five themes: emotional instability, transformation in nursing practice, resourcefulness in nursing practice, accepting and adjusting nursing roles, and spiritual belief and care.

### 3.1. Emotional Instability

This theme could focus on the internal conflicts and psychological challenges nurses faced in balancing their professional duties with personal concerns during the pandemic. The emotional strain from fear of exposure, uncertainty, and the burden of duty led to psychological and moral dilemmas affecting nurses' well-being and their approach to patient care.

#### 3.1.1. Psychological Strain

This unprecedented pandemic challenges the role of nurses in clinical settings. In the early stages of the pandemic, nurses were highly concerned about their health. Their primary concern was the risk of acquiring and bringing the infection home to their loved ones. Nurses were in a dilemma in fulfilling their duties and responsibilities. The worry of being infected and exposed to this new disease was aggravated by the lack of specific management and cure and the burden of fulfilling their Nightingale pledge of service regardless of the patient's health condition.

*“During the early stage of the pandemic, I was so worried that I would be infected. Sometimes I felt so paranoid that when I felt hot, I always thought I was infected already. Sometimes I cannot sleep thinking about what I have done during the day, always thinking if I encountered a patient with signs of COVID.”* Nurse 7

#### 3.1.2. Moral Conflict in Nursing Care

Nurses were caught in a difficult situation where they had to fulfill their duty to care for patients despite the fear of infection. The Nightingale Pledge, which calls for a nurse's service regardless of the patient's condition, became a source of internal conflict. Even with the risks involved, the overwhelming responsibility to care for patients created tension between professional obligations and personal safety concerns.

*“As a nurse, I found myself in a real dilemma when fulfilling my duties and responsibilities. The constant worry of being infected and potentially exposing my loved ones to this new disease was overwhelming. The situation was made worse by the lack of specific management or a cure, and yet, we still had the burden of honoring my pledge as a nurse, which is to cure patients. The worst is I experience sleep issues, making it even harder to cope.”* Nurse 9

#### 3.1.3. Compromised Care and Emotional Distancing

The participant mentioned that due to the fear of contracting the virus, many nurses struggled with providing emotional and psychological support to patients. The need to minimize contact led to the neglect of comfort measures, like physical touch and personal conversation, which nurses are typically trained to provide. This emotional distancing created a sense of guilt, as they could not offer the compassionate care they felt was necessary, leading to feelings of inadequacy.

*“I only give medication to my patient, I don't even talk to them since I am afraid I might be infected with the disease.”* Nurse 9

*“I don't provide holistic care anymore; it is more on physical care. I can't even talk to my patient, which makes me feel guilty since I know they are also struggling and need someone to talk to.”* Nurse 12

#### 3.1.4. Feeling of Lack of Security

Nurses also mentioned that they hesitated to care for a patient with COVID-19. They always felt a lack of security. They could not even provide physical touch and comfort to their patient. Psychological care was also neglected. They were providing care that merely involved drug administration.

*“During the early days, I did not want to stay in the room of a COVID-19 patient when I provided care. I entered a patient's room to give the due medications and turned the patient in if needed. We did not even talk to and comfort them because I always thought that if I stayed longer inside the patient's room, I would be exposed and infected. Yes, I always wear full PPE, but I still felt unsecured.”* Nurse 13

### 3.2. Learning, Relearning, and Unlearning Nursing Care

This theme encompasses how nurses navigated the uncertainty and evolving guidelines brought by the pandemic, using new knowledge, retraining, and revisiting old practices to ensure the best possible care for themselves, their patients, and their communities.

#### 3.2.1. Learning New Skills

COVID-19 is a new disease that sporadically affects humans. No specific management and cure were identified in the early stage of the disease, as nurses adapted and adjusted to disease management and control. Accordingly, training was instituted to enhance the skills of nurses. New skills were instituted to ensure that healthcare providers were protected. One of the trainings performed was learning new skills to prevent and spread the disease. Nurses had substantial training on the new guidelines and protocols being implemented.

*“There is no definitive care and management for COVID-19. Even the guidelines and protocols rapidly change over time. However, I appreciate that the administration is very supportive and regularly conducts training. Even the Ministry of Health (MOH) conducts a spot evaluation to ensure we follow the protocols and guidelines.”* Nurse 11

The participants mentioned that during the early stages of the pandemic, they turned to reliable online resources to better understand how to protect themselves from COVID-19 and to stay informed about the appropriate interventions for patients infected with the virus. This was crucial because, at the time, there was a significant amount of uncertainty surrounding the virus, and guidance from official sources was often evolving. Nurses sought trustworthy websites, medical journals, and guidelines from health organizations like the World Health Organization (WHO) and the Centers for Disease Control and Prevention (CDC) to stay updated on the latest safety protocols, protective equipment requirements, and best practices for treating COVID-19 patients.

*“Since some guidelines and protocols are confusing, I also read studies and information about COVID-19 online to ensure that I am updated.”* Nurse 8

*“I use reliable resources to learn about COVID. Using resources like the WHO and CDC, I better understood COVID-19 transmission, symptoms, and clinical management strategies.”* Nurse 13

#### 3.2.2. Relearning and Revisiting Basic Nursing Practices

Relearning and revisiting fundamental principles played a crucial role in helping nurses adapt to the evolving challenges of the pandemic, particularly regarding infection control. The crisis highlighted gaps in existing institutional practices and the need for continuous education, especially when faced with a novel and highly contagious disease like COVID-19. As the virus rapidly spread, healthcare institutions quickly recognized that their previous training and protocols needed to be updated and reinforced. As mentioned by one of the respondents:

*“This pandemic taught me to appreciate relearning the basic information that sometimes I forget since I have been working long in the hospital. Sometimes, if we practice, we modify the standard, which becomes a practice in the hospital. At least it is being corrected this time.”* Nurse 1

Relearning basic infection control was not just about knowledge but also about skill-building. Nurses enhanced their ability to identify potential infection risks, respond quickly to emerging symptoms, and safely handle contaminated equipment and materials. This knowledge also extended to self-care, as nurses learned how to recognize the early signs of infection in themselves and their colleagues and when to take preventive measures or seek medical attention.

*“Our hospital conducted a retraining on infection control, and I found that this training not only helped correct and improve the practices within our institution but also updated our skills. During the pandemic, continuous training and implementing new practices are very important for our safety and in providing the care we give to patients.”* Nurse 5

#### 3.2.3. Unlearning Previous Practices and Adjusting to New Standards

Unlearning is one of the standard practices that nurses also perform. Nurses mentioned that they had unlearned practices not correctly performed before the pandemic, such as not wearing a face mask when caring for patients with infectious and non-infectious diseases. Handwashing and hand sanitizer use have become religious, unlike before the pandemic. They also strived to improve health conditions by taking multivitamins, washing clothes worn during clinical duty, and soaking them with bleach. Nurses also performed proper handwashing techniques and followed the infection-control protocol more religiously than before the pandemic.

*“This pandemic made many changes to some of our hospital practices. Some standard practices were not correctly performed before the pandemic. I must admit that I did not follow proper handwashing when I washed my hands. What was important was that I washed my hands. Before, I just put my uniform in the laundry basket when I went home, but now I need to soak it with bleach. My practices have changed a lot. Sometimes I smiled and asked myself, Why did this pandemic have to happen for me to practice standard procedures?”* Nurse 3

### 3.3. Resourcefulness in Nursing Practice

This theme highlights how nurses demonstrated creativity, critical thinking, and resilience in response to the challenges posed by the COVID-19 pandemic, particularly in situations involving resource shortages, evolving protocols, and the need to ensure patient and staff safety. Nurses used their ingenuity to maximize limited resources, collaborate effectively with colleagues, and stay informed through various platforms, all while adapting to the rapidly changing healthcare environment.

#### 3.3.1. Maximizing Limited Resources

Nurses had to find ways to make do with scarce resources like PPE. This included reusing materials, rotating shifts for PPE use, and employing creative strategies to ensure the safety of both patients and staff. Nurses were resourceful during the pandemic, especially when PPEs were insufficient. Nurses need to utilize and maximize their resources.

*“This pandemic taught us to be resourceful. We have to utilize available resources and ensure we maximize their use. There was a time when PPE was limited. Those nurses assigned to provide care to patients with COVID-19 should wear full PPE. Those nurses providing care to a non-COVID patient will wear N95 face masks to save some PPE for the other shifts.”* Nurse 14

*“We have to save our PPE, so what we did was to let those nurses in non-contaminated patients wear N95, and those nurses on the COVID unit should wear full PPE.”* Nurse 5

#### 3.3.2. Collaboration and Problem-Solving

Nurses worked together to address challenges, updated one another on new guidelines, and helped each other navigate conflicting or changing protocols. Their teamwork and shared commitment to problem-solving were critical in maintaining care standards. The nurses mentioned that the shortage of essential PPE like masks, gloves, gowns, and face shields during the pandemic tested their ability to adapt, think critically, and make the most of limited resources, which was vital in ensuring that patient care continued despite the pandemic's many challenges. This resilience and creativity were key in ensuring the safety of healthcare workers and the quality of care delivered to patients during one of the most challenging periods in modern healthcare history.

*“We need to utilize and maximize the available resources creatively and effectively. For instance, when there was a lack of N95 masks, we resorted to reusing masks after following strict protocols for safe storage and disinfection, even though this was not ideal for optimal protection.”* Nurse 12

#### 3.3.3. Adapting to Changing Guidelines

Nurses needed to adjust quickly to new guidelines and protocols, particularly when resources were limited or unclear. Nurses worked together to implement the new guidelines and protocols. They were also very accommodating and willing to help their colleagues as needed. Their resourcefulness was observed in their ability to solve problems in their workplace, especially when protocols and guidelines were conflicting and rapidly changing.

*“What I observe in our workplace is that everyone helps each other. If there are new guidelines, those who are aware update others.”* Nurse 5

#### 3.3.4. Self-Directed Learning

Nurses took the initiative to seek reliable information from online sources like the WHO and CDC to stay updated on the best practices for patient care and self-protection. Nurses also use social media to learn more about patient management and self-protection.

*“I also read on some sites, like those of the WHO and CDC, how to provide care for the patient. These sites provide information and are very reliable sources.”* Nurse 3

#### 3.3.5. Leadership Management and Staff Support

Nurse leaders were pivotal in supporting their teams, ensuring adequate resources were provided, training was conducted, and prioritizing staff welfare. Nurse leaders were also beneficial. They ensured that the patients' welfare was always prioritized. These leaders conducted training and ensured that PPE resources were readily available. They also immediately conducted fit tests for masks to ensure correct fitting and protection.

*“The nurse leaders in our hospital were very supportive during this pandemic. Supplies were provided, especially the PPEs, which were absolutely and supremely important. The ever-changing protocols and guidelines were cascaded to us, and they trained us for any changes. If staff members were exposed and developed symptoms, necessary actions were taken by putting them in isolation. Necessary support was also provided throughout the entire course of quarantine.”* Nurse 8

### 3.4. Accepting and Adjusting Nursing Roles

This theme emphasizes how nurses adapted to their roles and took on extended responsibilities during the pandemic. Despite the challenges and fears, nurses accepted and adjusted to their new realities, providing comprehensive, holistic care beyond their typical scope. Their willingness to take on these expanded roles reflects a deep sense of duty, resilience, and commitment to patient well-being, even in uncertain and personal risk.

#### 3.4.1. Acceptance and Comfort in New Roles

Nurses became increasingly comfortable with their new responsibilities as they adjusted to the realities of caring for COVID-19 patients. They grew confident in their ability to provide care while following necessary safety protocols, which allowed them to offer more comprehensive patient care, both physically and emotionally. As nurses provided care to patients with COVID-19, they became comfortable and used to the routine care of patients as they accepted and adjusted to their role. All nursing-care aspects were covered. Nurses ensured they provided their patients with physical, emotional, and social care. They even stayed with their patient for extended hours. As claimed by one nurse:

*“Before, when we gave nebulization to our patient, we switched the machine and left the patient immediately. Now, we can switch on the machine while providing other care to the patient since we have already adjusted, and we now know that our PPE is one of the most effective ways to protect us.”* Nurse 2

*“I also observed that now, I can stay and talk to my patient and assess my patient properly as long as I am using proper PPE.”* Nurse 10

*“Now, I provide the care the patient needs without worrying that I will be infected, unlike before.”* Nurse 6

#### 3.4.2. Commitment to Duty

Despite the personal and professional challenges posed by the pandemic, nurses displayed an unwavering sense of duty. Their willingness to extend shifts and care for more patients, even when facing personal fears of infection or burnout, reflected their deep commitment to their roles and patients' needs. Furthermore, the nature of the pandemic required nurses to spend long hours with their patients, often beyond typical shift lengths. In some cases, the volume of patients and the care demands extended hours of physical presence. Many nurses chose to stay with patients longer, offering comfort and monitoring their condition continuously. This commitment, driven by duty and compassion, allowed nurses to provide comprehensive care and ensured patients were not left feeling abandoned or neglected, even during their most vulnerable moments.

*“During the pandemic, we stay longer in the hospital since we have lots of patients and we need to make sure that we need to provide care to them.”* Nurse 3

*“I was really hard during the pandemic even we are afraid to work since some of our colleagues are being infected. But looking to our patient, we opted to stay and work longer to ensure that we provide holistic care to our patient.”* Nurse 12

### 3.5. Living With COVID-19

Living with COVID-19 is about surviving the pandemic and how the participants have adapted and adjusted in their work during the pandemic. The key to successfully navigating COVID-19 was the adaptation and resilience of nursing in their practice. Living with COVID-19 shows that the participants develop a more sustainable approach to living with the virus, balancing safety with the return to normalcy over time.

#### 3.5.1. Adaptation in Nursing Practice

With time, nurses became aware of how to protect themselves. They provided care to a COVID-19 patient just like those without COVID-19. Nurses learned preventive measures while providing care. They are not even worried about being infected. As nurses mentioned, they learned to live with COVID-19, meaning they know how to manage a COVID-19 patient and protect themselves and their families. Providing care to a COVID-19 patient is conducted by nurses willingly and has become routine for them.

*“The fear that this pandemic brought to us front liners was unspeakable. When the virus was new, there were frequent changes in guidelines and protocols, and we were doubtful if this specific protocol would work. However, as the days went on, protocols were being established, and we got used to this virus, its nature, and its management. We got on our feet with the situation.”* Nurse 4

#### 3.5.2. Resilience in Nursing Practice

Resilience in nursing practice refers to a nurse's ability to adjust, recover, and maintain effective care despite challenging situations, stress, or adversity during the pandemic. It involves problem-solving skills, enabling nurses to manage workload and continue their professional duties under challenging circumstances.

*“In the beginning, it was exhausting and stressful because we were facing a new contagious disease, but as we get used to managing the situation, the patients' workload became manageable”* Nurse 5

### 3.6. Spiritual Belief and Care

This theme underscores the importance of spirituality in the lives of nurses during the pandemic, highlighting how their spiritual beliefs not only provided personal comfort and resilience but also influenced the way they delivered care to their patients. Nurses integrated their spiritual practices into their professional roles, using prayer and spiritual rituals as a means to cope with stress, find strength, and maintain hope during an overwhelming and uncertain time.

#### 3.6.1. Spiritual Practices as a Coping Mechanism

The participants mentioned that spirituality was a vital tool for managing the immense stress and emotional strain caused by the pandemic. Prayer, meditation, and other spiritual practices helped nurses find inner peace, reduce anxiety, and regain control in the face of overwhelming challenges. Most of the nurses mentioned that spiritual beliefs and care were not neglected from the start of this pandemic up to the present. This finding meant that nurses' spiritual beliefs and care were part of every aspect of their nursing care.

*“For me, the most important care that I can provide from the start of this pandemic up to date is my prayer. Because I believe that only God can help us in this pandemic.”* Nurse 4

*“I pray silently when I care for my patients that God will heal them and help them recover.”* Nurse 13

#### 3.6.2. Spirituality as a Source of Resilience

The spiritual connection to a higher power or belief system became a source of strength and resilience, allowing nurses to endure their work's emotional and physical demands. In a crisis, their faith helped them maintain hope and persevere through difficult moments, contributing to their ability to continue delivering compassionate care.

*“This pandemic is very stressful, but praying provides comfort to me and helps me to reduce my anxiety.”* Nurse 7

*“Sometimes, if I can't handle my stress, I pray and call my family back home to pray for my safety,”* Nurse 15

## 4. Discussion

This study explored the experiences of staff nurses in providing nursing care during the COVID-19 pandemic and how it evolved to adapt to the pandemic situation. The findings revealed six major themes that describe how nursing care adapted throughout the pandemic experiences of nurses.

In the first theme, nurses initially experienced emotional instability. This result was similar to Lai et al.'s findings that nurses experience psychological challenges such as anxiety, stress, and depression when caring for COVID-19 patients [25]. Studies in Iran and China have also shown that nurses experience psychological challenges, such as anxiety related to separation from family and fear of infecting them. In the early stage of the pandemic, nurses experienced emotional instability [7, 25]. Another study conducted among nurses from Spain reported that nurses working in COVID-19 units experienced high levels of emotional fatigue and anxiety [26]. Additionally, a phenomenological study in Canada found that nurses faced emotional challenges, including stress, anxiety, exhaustion, frustration, guilt, and loneliness, which were exacerbated by uncertainty during the pandemic [27]. Nurses experienced emotional instability during the COVID-19 pandemic due to overwhelming workloads, moral distress, and inadequate organizational support [28]. The surge in patient numbers led to physical exhaustion and emotional fatigue while witnessing high patient mortality and working with limited resources intensified their anxiety and stress [29]. Additionally, the findings of this study suggest that the lack of pandemic preparedness, including insufficient protective equipment and unclear guidelines, left many nurses feeling unprepared and unsupported, further exacerbating their emotional struggles. As expressed by nurses, this emotional turmoil often led to sleep disturbances, heightened anxiety, and a reluctance to provide holistic patient care beyond basic medical tasks, reflecting their struggle to balance their safety with their commitment to patient well-being. These consistent findings of this study, with studies across different countries, underscore the global impact of the pandemic on nurses' mental health.

Nurses then started learning, relearning, and unlearning nursing practices in the second theme. This study's findings showed that nurses acknowledged the importance of learning new skills that are effective in the pandemic, relearning previous nursing skills, and unlearning those ineffective institutional practices for the prevention of spread and protection from COVID-19. As Sun et al. mentioned, the COVID-19 pandemic allows nurses to learn new skills that help them grow professionally [2]. Rathnayake et al. also mentioned that nurses use various learning strategies during the pandemic to cope with the demand for care provided to COVID-19 patients [30]. Also, a previous study argued that crises stimulated learning and unlearning in various levels of organizations, which is necessary to ensure effective crisis management [31]. In particular, the rapidly changing clinical environment during the pandemic required nurses to stay updated with evolving guidelines and protocols, fostering an adaptive learning culture. For instance, nurses had to unlearn routine procedures no longer considered safe and adopt evidence-based practices to minimize infection risks. Studies like those by Dewey et al. and Adams and Walls emphasized the importance of institutional support in promoting continuous learning and competency development among healthcare workers in times of crisis [32, 33]. Moreover, crises like the COVID-19 pandemic stimulate learning and unlearning at various organizational levels, which is essential for effective crisis management and resilience [34]. Through this dynamic process, nurses enhanced their clinical competence and contributed to improving healthcare delivery systems, ensuring patient safety and quality care amidst unprecedented challenges.

The lack of resources is a problem nurses encounter, but this pandemic taught them to be resourceful. This finding contradicted studies stating that nurses cannot provide adequate care due to lacking PPE and resources [2, 25, 30]. A previous study argued that the lack of resources during the pandemic often made nurses decide about their care based on the available resources [32]. The present results showed that the pandemic taught nurses to be resourceful. Thus, they devised a strategy in which nurses providing care to COVID-19 patients were required to wear full PPE, while those caring for non-COVID-19 patients wore only N-95 masks only. This practice may seem inappropriate, but it is one of the best ways to maximize the available resources.

The fourth theme was accepting and adjusting to the nursing roles during the pandemic. Nurses eventually accepted and adjusted to their nursing role in providing care to COVID-19 patients. This finding may be associated with nurses' obligation, motivation, and the Nightingale pledge to provide care to a patient without discrimination. Similarly, studies conducted in Australia [33], South Korea [11], and China [2] have shown that professional obligation, commitment, and dedication are reasons for nurses to provide care to a CoV-infected patient. Furthermore, Galehdar et al. found that nurses providing care to patients because of the Nightingale pledge believe that providing care is just like a mother who would endanger herself to protect her children [7]. Improving resiliency during a pandemic is also a contributory factor in accepting the role of nurses [6]. A previous study reported on the adaptation process of nurses during the COVID-19 pandemic to provide care to patients. It noted that the core concept in the nurses' adaptation process was becoming proficient nurses alongside their colleagues during difficult times [35]. This signifies the importance of growing together as nurses, and adjusting together to the roles necessary to combat the pandemic.

Nurses further eventually learned to live with COVID-19. Provision of care became a routine. Nurses did not think of being infected or experiencing anxiety because they became aware of their role in preventing and controlling COVID-19. According to Sun et al., nurses learn to love, respect, and honor their profession during this pandemic, owing to the improvement and professional development gained from their experience caring for a COVID-19 patient [2]. Also, a related qualitative study showed that nurses during the pandemic experienced professional growth as they went through the pandemic times with the support and camaraderie of their colleagues [35]. Nurses also learned to live with COVID-19 because of the support of management and authorities [7].

Interestingly, it was found that spiritual beliefs and practices were very evident during the pandemic as factors that support resilience, mutual support, mental health, and well-being of nurses. Nurses always remember their spiritual beliefs and care to help others overcome stressful situations during the pandemic [4]. Similarly, Rathnayake et al. found that nurses' spiritual beliefs and practices are the most effective coping mechanism during the COVID-19 pandemic [31]. These religious beliefs help individuals overcome stressful situations and psychological problems [4, 36, 37]. An integrative review reported that healthcare providers' spirituality helps them cope during the pandemic and is beneficial to their mental health and well-being amidst their harrowing experiences in the COVID-19 pandemic. The same review also showed that addressing the spirituality of healthcare providers during the pandemic resulted in greater patient satisfaction with the care they provide [37].

### 4.1. Limitations of the Study

The study's limitation includes the qualitative method used, which limits the generalization of the results. Although qualitative research does not aim for statistical generalizability, the insights gained provide depth and context to inform practice and policy in similar healthcare environments. Another limitation of the study is the sampling strategy, in which the majority of the nurses interviewed was not native to Saudi Arabia and may not have consistent perspectives with people who are from the Kingdom. This raises concerns about the generalizability of the findings across different cultural groups. Thus, future researchers should focus on conducting a similar study on Saudi nurses. Also, readers should consider the characteristics of the nurses included in this study (i.e., predominantly Filipinos) when interpreting the findings. The recruitment of participants began through recommendations from the researcher's colleagues and friends. While this provided a good starting point for identifying potential participants, and the researcher exercised due diligence in selecting them based on these recommendations, there is still a possibility of bias from this recruitment method. Therefore, readers should consider this when interpreting the study's findings. Another limitation is the use of the purposive sampling technique. The findings may not reflect the experience of the nurses who cared for COVID-19 patients in the country. Further, the use of both face-to-face and Zoom interviews may have led to inconsistencies in data quality. Specifically, the depth and richness of responses may have varied between formats, with face-to-face interviews potentially encouraging more detailed responses due to increased rapport and non-verbal communication. In contrast, while efficient, Zoom interviews may have limited participants' emotional nuance and comfort, possibly affecting their answers' completeness. While no significant differences in consistency were observed, the limitations of the virtual interview format, including technical difficulties and reduced rapport-building opportunities, were potential factors influencing the data quality.

### 4.2. Implications to Nursing Management

This qualitative study has valuable implications for ensuring the highest level of nursing care during health crises, such as the COVID-19 pandemic. The findings described the changes in nursing care, elucidating the different areas requiring adjustment and adaptation in providing nursing care during a pandemic. The findings on the adjustments in nursing care in this pandemic can also be used to uncover, discover, and re-discover the strengths and weaknesses of nurses as they provide nursing care to patients during health crises. Hospital policymakers can use the results to create policies and interventions and thus ensure that nurses are providing the best quality of care to patients during a health crisis, such as the COVID-19 pandemic. For instance, we found that the emotional instability of nurses in the early stages of the pandemic can be used to create emotional support programs for nurses for immediate implementation at the start of a health crisis and follow through for its entire duration.

The finding focused on the learning, relearning, and unlearning of nursing care signifies the critical role of nurses' continuous education, especially during health crises, to ensure they are properly equipped with the knowledge and skills to care for affected patients. This finding emphasizes that nursing management must create a dynamic, supportive, and resource-rich environment that promotes lifelong learning and adapts to the evolving needs of nursing care, particularly during health crises. Further, nursing managers should establish mechanisms for regular assessment of clinical skills and competencies. This could involve simulation-based training, scenario drills, and competency evaluations to ensure nurses are aware of new guidelines and can apply them effectively under pressure.

The emphasis given by this study on the importance of nurses' spirituality also supports the need to create spirituality-centered programs in hospitals during health crises. The importance of spirituality as a positive coping mechanism among nurses during the pandemic signals the need to ensure a workplace that has a positive spiritual climate. Hospital managers should create a work climate that supports and promotes spirituality among nurses and other healthcare providers. Spiritual support and interventions can be implemented to support health workers during health crises, such as the pandemic. Areas in the hospital should be created for designated spiritual and religious spaces where health providers can safely perform their spiritual and religious practices, as these are part of their coping mechanisms during difficult situations. The spaces should accommodate individuals of various spiritual and religious backgrounds.

Furthermore, this study showed that resource allocation and rationing issues exist in nursing practice during pandemics and that nurses contribute to alleviating the negative effects of inadequate resources by being resourceful, accepting and adjusting their roles, and learning to live with the pandemic. These strengths of nurses should be exemplified during health crises. However, healthcare facilities also need to guarantee adequate resources to avoid problems and ensure the quality of nursing care without sacrificing the welfare of healthcare professionals, such as nurses.

## 5. Conclusions

This qualitative thematic-approach study explored the nursing care for patients infected with COVID-19, and how it adapted to the situations during the pandemic. It was uncovered that nurses experienced emotional instability during the pandemic. They experienced fear and worry, particularly during the earlier phase of the pandemic. The pandemic also provided opportunities for nurses to learn new knowledge and skills on COVID-19 prevention, protection, and intervention. They relearned basic knowledge of infection control and unlearned nursing practices that were not correctly performed before the pandemic. They developed their resourcefulness during the pandemic to cope with inadequate resources. They needed to accept and adjust their nursing roles to provide holistic patient care, thereby learning how to live with COVID-19. Finally, this study provided additional evidence of the critical role of nurses' spirituality in changing nursing care during the COVID-19 pandemic. The nurse's spirituality was an important component of their coping mechanisms during the difficult times of the pandemic.

## Figures and Tables

**Table 1:** Demographic profile of the study participants.

Participant	Age	Gender	Years of experience	Nationality	Highest educational attainment
Nurse 1	32	Male	11	Filipino	Masters
Nurse 2	35	Male	8	Filipino	Masters
Nurse 3	28	Female	5	Indian	BSN
Nurse 4	29	Male	6	Filipino	BSN
Nurse 5	27	Male	2	Saudi	BSN
Nurse 6	34	Female	6	Filipino	BSN
Nurse 7	29	Male	5	Saudi	BSN
Nurse 8	33	Male	8	Filipino	BSN
Nurse 9	31	Female	8	Saudi	Masters
Nurse 10	35	Male	13	Filipino	BSN
Nurse 11	40	Female	20	Filipino	BSN
Nurse 12	30	Female	8	Filipino	BSN
Nurse 13	34	Male	9	Filipino	BSN
Nurse 14	32	Male	10	Filipino	BSN
Nurse 15	38	Female	15	Filipino	BSN

## Data Availability

The data that support the findings of this study are available from the corresponding author upon reasonable request.
